# Heme oxygenase-1 promotes tumor progression and metastasis of colorectal carcinoma cells by inhibiting antitumor immunity

**DOI:** 10.18632/oncotarget.4075

**Published:** 2015-05-28

**Authors:** Geom Seog Seo, Wen-Yi Jiang, Jin Hua Chi, Hao Jin, Won-Chul Park, Dong Hwan Sohn, Pil-Hoon Park, Sung Hee Lee

**Affiliations:** ^1^ Digestive Disease Research Institute, Wonkwang University College of Medicine, Jeonbuk, Republic of Korea; ^2^ Institute of Pharmaceutical Research and Development, College of Pharmacy, Wonkwang University, Jeonbuk, Republic of Korea; ^3^ College of Pharmacy, Yeungnam University, Gyeongbuk, Republic of Korea

**Keywords:** HO-1, colorectal carcinoma cell, antitumor immunity, ICAM-1, CXCL10

## Abstract

Heme oxygenase-1 (HO-1) is upregulated in colorectal carcinoma (CRC) cells. However, the role of HO-1 in the metastatic potential of CRC remains to be elucidated. In this study, we investigated the potential of HO-1 to control the antitumor immunity of CRC. Intercellular adhesion molecule-1 (ICAM-1) plays an important role in the immune surveillance system. Hemin-induced HO-1 expression suppressed the expression of ICAM-1 in human CRC cells. HO-1 regulated ICAM-1 expression via tristetraprolin, an mRNA-binding protein, at the posttranscriptional level in CRC cells. The upregulated HO-1 expression in CRC cells markedly decreased the adhesion of peripheral blood mononuclear lymphocytes (PBMLs) to CRC cells and PBML-mediated cytotoxicity against CRC cells. Production of CXCL10, an effector T cell-recruiting chemokine, was significantly reduced by the increased HO-1 expression. The expression of the CXCL10 receptor, CXCR3, decreased significantly in PBMLs that adhered to CRC cells. HO-1 expression correlated negatively, although nonsignificantly, with ICAM-1 and CXCL10 expression in xenograft tumors. Taken together, our data suggest that HO-1 expression is functionally linked to the mediation of tumor progression and metastasis of CRC cells by inhibiting antitumor immunity.

## INTRODUCTION

Colorectal carcinoma (CRC) contributes to the overall cancer mortality [1]. Although mortality can be prevented by surgical resection before tumor cell dissemination, CRC progression and metastases are the main causes of death [2]. Therefore, elucidation of the molecular pathways involved in tumor progression and metastasis may yield novel therapeutic approaches.

Heme oxygenase-1 (HO-1) represents a key biological molecule in the adaptive response to cellular stress. Although the overexpression of HO-1 may exert beneficial effects in a number of pathological conditions, a growing body of evidence suggests that HO-1 plays a role in the pathogenesis and progression of several types of malignancy [3]. HO-1 expression and activity are elevated in various tumors and its expression is usually higher in cancer tissue than in the surrounding healthy tissues [4–7]. However, the results of studies into the role of HO-1 in tumor progression remain controversial. Some observations suggest that elevated levels of this protein are associated with neoplastic growth. HO-1 could be a survival factor for CRC cells because it inhibits apoptosis, which prolongs cell survival during the multiple mutations involved in colon carcinogenesis [8–10]. In addition, HO-1 is commonly regarded as a potent proangiogenic enzyme. Angiogenesis is critical for both tumor growth and metastasis. Thus, the proangiogenic action of HO-1 may further support tumor progression [11, 12]. By contrast, some studies have suggested that HO-1 induction contributes to a lower risk of lymph node metastasis in human colorectal and oral carcinoma [13]. Therefore, the role of HO-1 in the metastatic potential of CRC cells remains to be elucidated fully.

The progression and metastasis of a malignant tumor are mediated by various factors present in the microenvironment. Among them, the escape of malignant cells from local and systemic immune control leads to the subsequent invasion of and metastases to distant organs. Thus, once a tumor cell escapes recognition by the immune surveillance system, it may acquire additional metastatic properties [14]. Cellular adhesion mediated by various membrane-associated adhesion molecules is essential for the proper function of immunological processes. Intercellular adhesion molecule-1 (ICAM-1) is known to play an important role in the interaction between tumor cells and host cytotoxic effector cells [15–17].

Immune cells are found in human solid tumors, and for a large array of primary tumors, the immune pattern of the tumor microenvironment is a major predictor of patient survival [18, 19]. High densities of T cells with a Th1 and CD8^+^ T cell cytotoxic orientation or of mature dendritic cells are essential for antitumor immunity [20–22]. By contrast, tumor infiltration by regulatory T cells (T_reg_) predicts a poor outcome [23–25]. Chemokines and their respective receptors are critical for T cell migration and homing [26–29]. High levels of CCL5/RANTES (a CCR5 ligand) and CXCL9/MIG and CXCL10/IP10 (ligands for CXCR3) in tumor tissues are associated with increased infiltration of cytotoxic effector T cells (T_eff_) [30–32]. By contrast, a high level of CCL22/MDC, the CCR4 ligand that preferentially attracts T_reg_, is associated with reduced survival in ovarian cancer [33].

Impairment of immune surveillance contributes to metastasis and poor clinical outcome. The purpose of this study was to investigate the effects of HO-1 in CRC cells with a special focus on the antitumor immunity of CRC.

## RESULTS

### HO-1 negatively regulates the TPA-induced ICAM-1 expression in CRC cells

**Figure 1:**
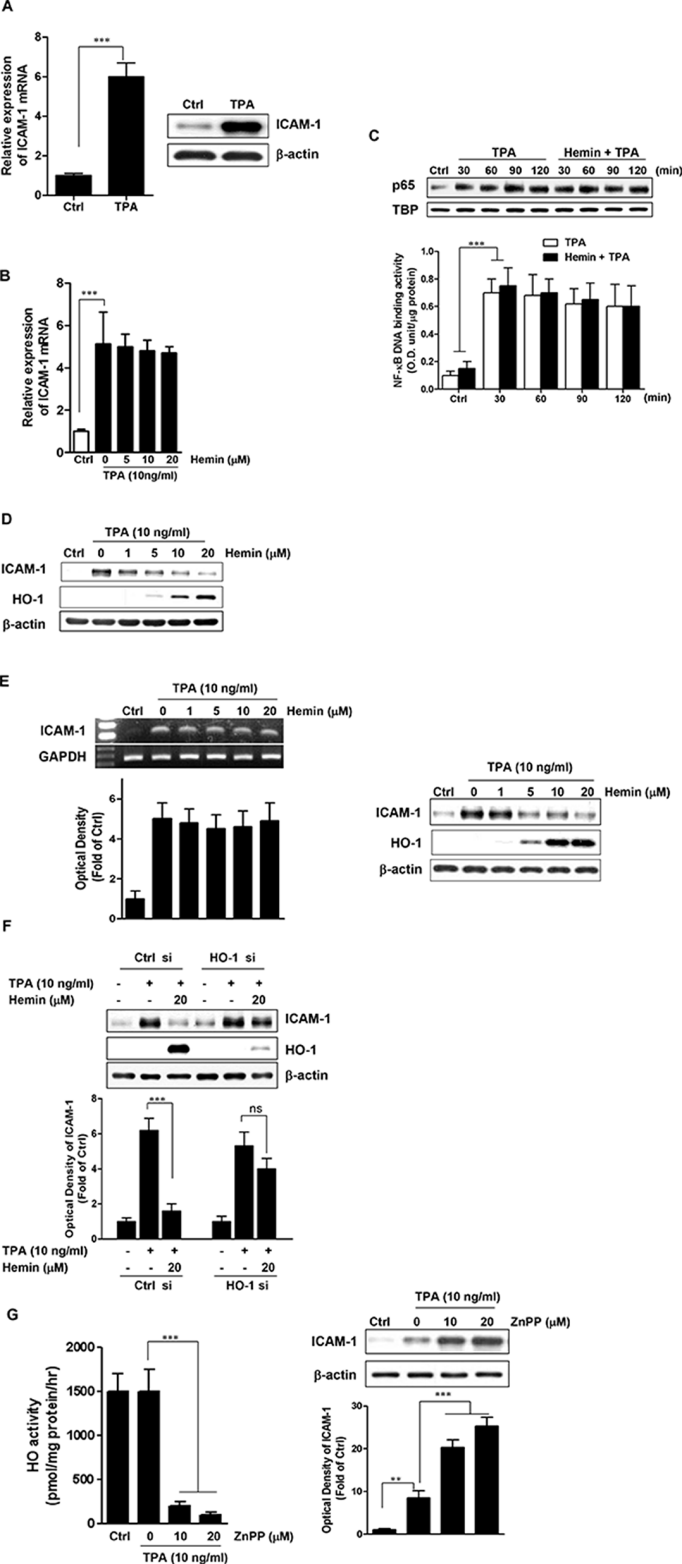
Effect of HO-1 on TPA-induced ICAM-1 expression **A.** HT-29 cells were treated with TPA (10 ng/ml) and then harvested for ICAM-1 mRNA (*left panel*) and ICAM-1 protein (*right panel*), which was measured using real time PCR and western blot analysis, respectively. The values represent mRNA levels normalized to that of GAPDH mRNA. **B.** The effect of HO-1 on TPA-induced ICAM-1 mRNA expression in HT-29 cells. The cells were pretreated with 0–20 μM hemin for 1 h followed by stimulation with 10 ng/ml TPA for an additional 10 h after which the cells were harvested for RNA preparation. RNA samples were analyzed by real time PCR to determine the levels of ICAM-1 mRNA. The values represent mRNA levels normalized to that of GAPDH mRNA. **C.** The effect of HO-1 on the p65 intranuclear translocation (*upper panel*) and on the NF-κB DNA binding activity (*lower panel*) in TPA-stimulated HT-29 cells. HT-29 cells were pretreated with or without 20 μM hemin for 1 h followed by incubation with 10 ng/ml TPA for the indicated intervals. The cells were harvested and the nuclear levels of NF-κBp65 and NF-κB DNA binding activity were determined by western blot and DNA binding assay, respectively. **D.** The effect of HO-1 on TPA-induced ICAM-1 protein expression in HT-29 cells. The cells were pretreated with 0–20 μM hemin for 1 h and were subsequently stimulated with 10 ng/ml TPA for an additional 24 h. The cells were harvested for ICAM-1 protein expression by western blot analysis. **E.** The effects of HO-1 on TPA-induced ICAM-1 mRNA and protein expression in Caco-2 cells. The cells were pretreated with 0–20 μM hemin for 1 h and were subsequently stimulated with 10 ng/ml TPA. The cells were harvested for ICAM-1 mRNA (*left panel*) and protein (*right panel*) expression by RT-PCR and western blot analysis, respectively. **F.** The effect of HO-1 silencing on the hemin-mediated reduction of ICAM-1 in TPA-induced HT-29 cells. HT-29 cells were transfected with siRNA against HO-1 (HO-1 si) or control siRNA (Ctrl si). After pretreatment with 20 μM hemin, and then stimulated with 10 ng/ml TPA for an additional 24 h. The expression levels of ICAM-1 and HO-1 were analyzed by western blot analysis. **G.** The effect of ZnPP on HO-1 activity (*left panel*) and TPA-induced ICAM-1 protein expression (*right panel*) in HT-29 cells. The cells were pretreated with 0–20 μM ZnPP for 1 h, followed by stimulation with 10 ng/ml TPA for an additional 24 h. The cells were harvested for measurement of HO activity and western blot analysis. The results were repeated in at least three independent experiments. The data are expressed as mean ± SD. ^**^P < 0.01 and ^***^P < 0.001. ns, not significant.

12-*O*-tetradecanoylphorbol-13-acetate (TPA) acts as a tumor promoter that induces the expression of ICAM-1 in many types of cells [34, 35]. Therefore, we first examined the effect of TPA on the expression of ICAM-1 in highly metastatic HT-29 cells. As shown in Figure 1A, TPA treatment increased both the mRNA (*left panel*) and protein (*right panel*) levels of ICAM-1 in HT-29 cells when assessed by real time PCR and western blot analysis, respectively. To identify the role of HO-1 in the ICAM-1 mRNA expression, hemin, an HO-1 chemical inducer, was used. As shown in Figure 1B, hemin did not alter the ICAM-1 mRNA expression in TPA-stimulated HT-29 cells.

Because the expression of ICAM-1 by TPA requires the transcription factor NF-κB, we examined whether HO-1 regulates NF-κB activation in TPA-stimulated HT-29 cells. We measured the effects of hemin on the p65 nuclear translocation and NF-κB DNA-binding activity [36, 37]. Hemin did not inhibit the nuclear localization of p65 protein induced by TPA-stimulation in HT-29 cells (Figure 1C, *upper panel*) and did not influence the TPA-induced NF-κB-dependent transcriptional activity (Figure 1C, *lower panel*). By contrast, treatment of the cells with hemin dose-dependently suppressed ICAM-1 protein expression induced by TPA (Figure 1D).

To verify the effects of HO-1 on ICAM-1 expression in CRC cells, we used another human CRC cell line, Caco-2 cells. Consistent with the results in HT-29 cells, hemin treatment affected the protein level but not the mRNA level of ICAM-1 (Figure 1E). These findings suggested that HO-1 regulates ICAM-1 expression at the posttranscriptional level in CRC cells. Examination of the cytotoxicity of hemin using an MTT assay indicated that hemin did not affect the viability of either type of CRC cell, even at a concentration of 20 μM (data not shown).

To confirm the effects of HO-1 on the hemin-mediated reduction of ICAM-1 expression, HT-29 cells were transfected with siRNA against HO-1. As shown in Figure 1F, hemin failed to reduce TPA-induced ICAM-1 expression in HO-1 siRNA-transfected cells. We also examined the effects of ZnPP, a competitive inhibitor of HO-1, on ICAM-1 expression in TPA-stimulated HT-29 cells. ZnPP abolished cellular HO activity in this experimental condition (Figure 1G, *left panel*). Interestingly, under the same experimental condition, ZnPP induced ICAM-1 expression beyond that induced by TPA alone (Figure 1G, *right panel*). These results suggested that the expression of ICAM-1 is critically dependent on HO-1.

### HO-1 induces destabilization of ICAM-1 mRNA

**Figure 2:**
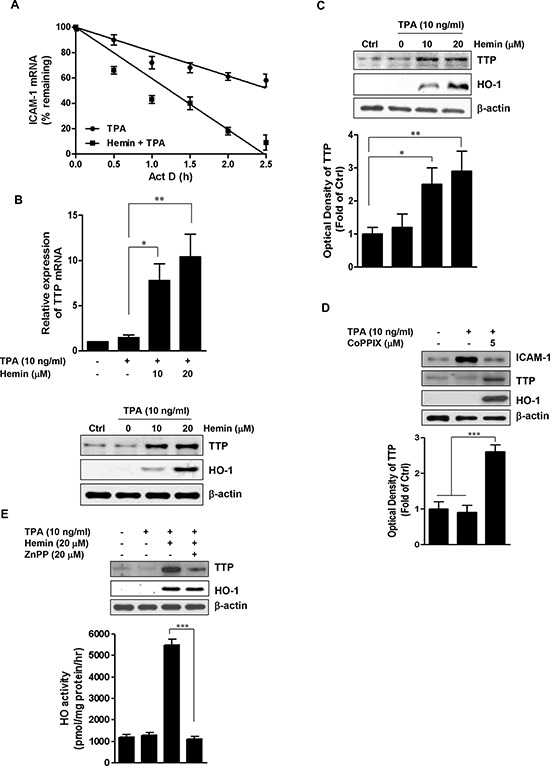
Effect of HO-1 on the stability of ICAM-1 mRNA and TTP expression **A.** HT-29 cells were treated with or without 20 μM hemin for 8 h. The cells were then stimulated with 10 ng/ml TPA for 2 h followed by the addition of 5 μg/ml actinomycin (Act D) for up to 150 min. ICAM-1 mRNA was measured using real time PCR. The expression levels were normalized to ICAM-1 mRNA levels. The normalized level of ICAM-1 mRNA at time 0 was set at 100%, and all other normalized mRNA levels were graphed relative to that value. Each point represents the mean ± SD from three independent experiments. **B.** Hemin-induced expression of TTP. HT-29 cells were pretreated with 10 and 20 μM hemin for 1 h followed by stimulation with 10 ng/ml TPA for an additional 16 h, TTP mRNA was quantified by real time PCR (*upper panel*). Values represent TTP mRNA normalized to GAPDH mRNA. Immunoreactive TTP was measured by western blot (*lower panel*). **C.** The effects of hemin on TTP protein expression in Caco-2 cells. Caco-2 cells were pretreated with 10 and 20 μM hemin for 1 h followed by stimulation with 10 ng/ml TPA and TTP prtoein was analyzed by western blot. **D.** HT-29 cells were treated with 5 μM CoPPIX for 1 h followed by stimulation with 10 ng/ml TPA and TTP prtoein was analyzed by western blot. **E.** HT-29 cells were pretreated with 20 μM ZnPP for 30 min, followed by 20 μM hemin for 1 h and further stimulated with 10 ng/ml TPA. The cells were harvested for western blot analysis and measurement of HO activity. A representative immunoblot out of three independent experiments is shown. Results were repeated in at least three independent experiments. The data are expressed as mean ± SD. ^*^P < 0.05, ^**^P < 0.01 and ^***^P < 0.001.

The results described above indicated that hemin can disrupt the posttranscriptional regulation of TPA-stimulated ICAM-1 expression. Because ICAM-1 has an AU-rich element (ARE) in its mRNA, we examined whether hemin could regulate the stability of ICAM-1 mRNA [38]. Act D was applied to inhibit the transcription following TPA stimulation, total cellular RNA was harvested after 0, 30, 60, 90, 120, and 150 min, and ICAM-1 mRNA levels were measured at these different times. The half-life of ICAM-1 mRNA was assessed by real-time PCR and found to be 156 min in TPA-only-treated cells (Figure 2A). Exposure to hemin markedly shortened the half-life to 74 min. This result indicated that HO-1 destabilized ICAM-1 mRNA in the TPA-stimulated HT-29 cells.

It has been reported that TTP promotes the destabilization of ARE-containing mRNAs [39, 40]. We next evaluated the effects of hemin on TTP expression in HT-29 cells. Hemin treatment significantly increased the induction of TTP mRNA (Figure 2B, *upper panel*) and protein (Figure 2B, *lower panel*) in a dose-dependent manner. Hemin treatment also caused a similar effect on TTP expression in Caco-2 cells (Figure 2C). We tested the effects of CoPPIX, another well-known inducer of HO-1, on the expression of ICAM-1 and TTP in TPA-stimulated HT-29 cells. Consistent with the results using hemin, CoPPIX significantly inhibited the TPA-induced ICAM-1 protein expression and significantly increased TTP protein expression (Figure 2D).

To determine whether HO-1 activity contributed to this upregulation of TTP, we also examined the effect of ZnPP on the hemin-induced upregulation of TTP. As shown in Figure 2E (*upper panel*), ZnPP blocked the upregulation of TTP by hemin without affecting the hemin-induced expression of HO-1 protein. Whereas the induction of HO-1 protein by hemin was accompanied by a marked increase in HO-1 activity, ZnPP abolished the cellular HO-1 activity in the presence of hemin (Figure 2E, *lower panel*). Thus, the inhibition of HO-1 activity by ZnPP blocked the hemin-induced upregulation of TTP. These results suggested that HO-1-induced TTP expression mediates the reduction in ICAM-1 expression in TPA-stimulated CRC cells.

### HO-1 expression in CRC cells inhibits PBML adhesion and PBML-mediated cytotoxicity of CRC cells

**Figure 3:**
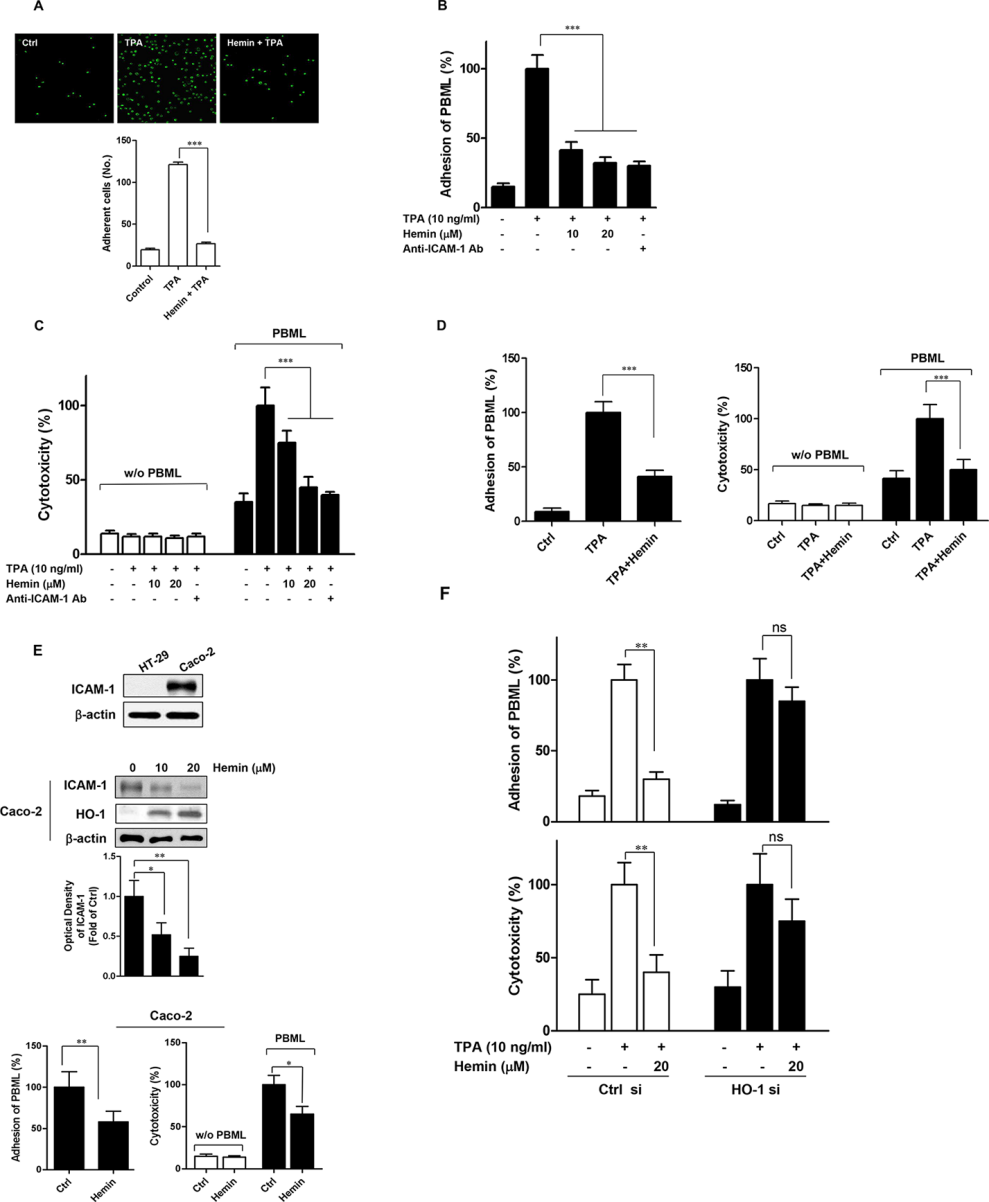
Effects of HO-1 on the PBML adhesion to and PBML-mediated cytotoxicity against CRC cells **A.** The effect of HO-1 on TPA-stimulated HT-29 cell adhesion to FBS-coated plastic wells. Fluorescent-labeled HT-29 cells were pre-incubated with 20 μM hemin for 30 min and then plated on FBS-coated plastic wells for 4 h in the presence of 10 ng/ml TPA. **B.** and **C.** The effect of HO-1 on the adhesion of PBMLs to HT-29 cells (B) and PBML-mediated cytotoxicity against HT-29 cells (C). HT-29 cells were pretreated with or without hemin for 1 h followed by incubation with 10 ng/ml TPA for 16 h prior to the assay. An anti-ICAM-1 antibody was used as a positive control. PBMLs were added to the HT-29 cells and cocultured at 37 °C for 1 h and 16 h for the PBML adhesion assay and HT-29 cytotoxicity assay, respectively. **D.** The effect of HO-1 on the adhesion of PBMLs to and PBML-mediated cytotoxicity against Caco-2 cells. PBMLs were added to the Caco-2 cells and cocultured at 37 °C for 1 h and 16 h for the PBML adhesion assay (*left panel*) and Caco-2 cytotoxicity assay (*right panel*), respectively. **E.** Basal ICAM-1 expression on HT-29 and Caco-2 cells. Each cell was plated and incubated for 24 h. ICAM-1 protein expression assessed by western blot analysis (*upper panel*). The effect of HO-1 on ICAM-1 protein expression in Caco-2 cells. The cells were treated with 10 and 20 μM hemin for 24 h. The cells were harvested for ICAM-1 protein expression by western blot analysis (*middle panel*). The effect of HO-1 on the adhesion of PBMLs to and PBML-mediated cytotoxicity against Caco-2 cells in the absence of TPA (lower panel). **F.** Hemin-mediated inhibition of adhesion of PBML to HT-29 cells and PBML-induced cytotoxicity against HT-29 cells were attenuated by transfection with HO-1 siRNA. HT-29 cells were transfected with siRNA against HO-1 (HO-1 si) or control siRNA (Ctrl si). After these transfected cells were incubated with TPA alone or TPA and hemin for 24 h, PBMLs were added and cocultured for 1 h and 16 h for the PBML adhesion assay and HT-29 cytotoxicity assay, respectively. The results were repeated in three independent experiments. Data are expressed as mean ± SD. *P* < 0.05, ^**^P < 0.01 and ^***^P < 0.001. ns, not significant.

Following our studies of the inhibition of ICAM-1 protein expression by HO-1 in TPA-stimulated CRC cells, we next evaluated the functional significance of this finding. We assessed the adhesion of HT-29 cells to FBS-coated plastic wells and found that this was significantly inhibited by hemin (Figure 3A). Because ICAM-1 has been reported to play an important role in the interaction between tumor cells and host cytotoxic effector cells [15–17], we evaluated the effects of HO-1 on the adhesion of PBMLs to CRC cells. As shown in Figure 3B, hemin inhibited the PBML adhesion to HT-29 cells in a dose-dependent manner.

We assessed the effects of HO-1 on PBML-mediated cytotoxicity against HT-29 cells and found that this was also significantly decreased by hemin compared with that in the TPA-stimulated cells (Figure 3C). Under the same experimental conditions, PBML adhesion and PBML-mediated cytotoxicity were significantly inhibited by an anti-ICAM-1 neutralizing antibody. This result demonstrated a direct involvement of ICAM-1 in PBML adhesion to and PBML-mediated cytotoxicity against HT-29 cells. Hemin treatment also inhibited PBML adhesion to and PBML-mediated cytotoxicity against Caco-2 cells (Figure 3D).

Immunoblot analysis indicated that ICAM-1 was constitutively expressed at a higher level in Caco-2 cells than in HT-29 cells (Figure 3E, *upper panel*). To verify the relationship between HO-1 and ICAM-1, we used Caco-2 cells without TPA. Treatment of Caco-2 cells with hemin dose-dependently suppressed ICAM-1 protein expression (Figure 3E, *middle panel*) and inhibited PBML adhesion to and PBML-mediated cytotoxicity against Caco-2 cells without TPA (Figure 3E, *lower panel*). These findings suggested that HO-1 inhibited ICAM-1 expression in the absence of TPA. To confirm the effect of HO-1 on these hemin-mediated effects, HT-29 cells were transfected with siRNA against HO-1. As shown in Figure 3F, the effects of hemin on the adhesion of PBML and PBML-mediated cytotoxicity of HT-29 cells were attenuated in HO-1 siRNA-transfected cells. These results suggested that HO-1 is required for the hemin-mediated inhibition of adhesion of PBMLs to CRC cells and PBML-mediated cytotoxicity against CRC cells.

### HO-1 modulates the T_eff_-recruiting chemokine expression in CRC cells

**Figure 4:**
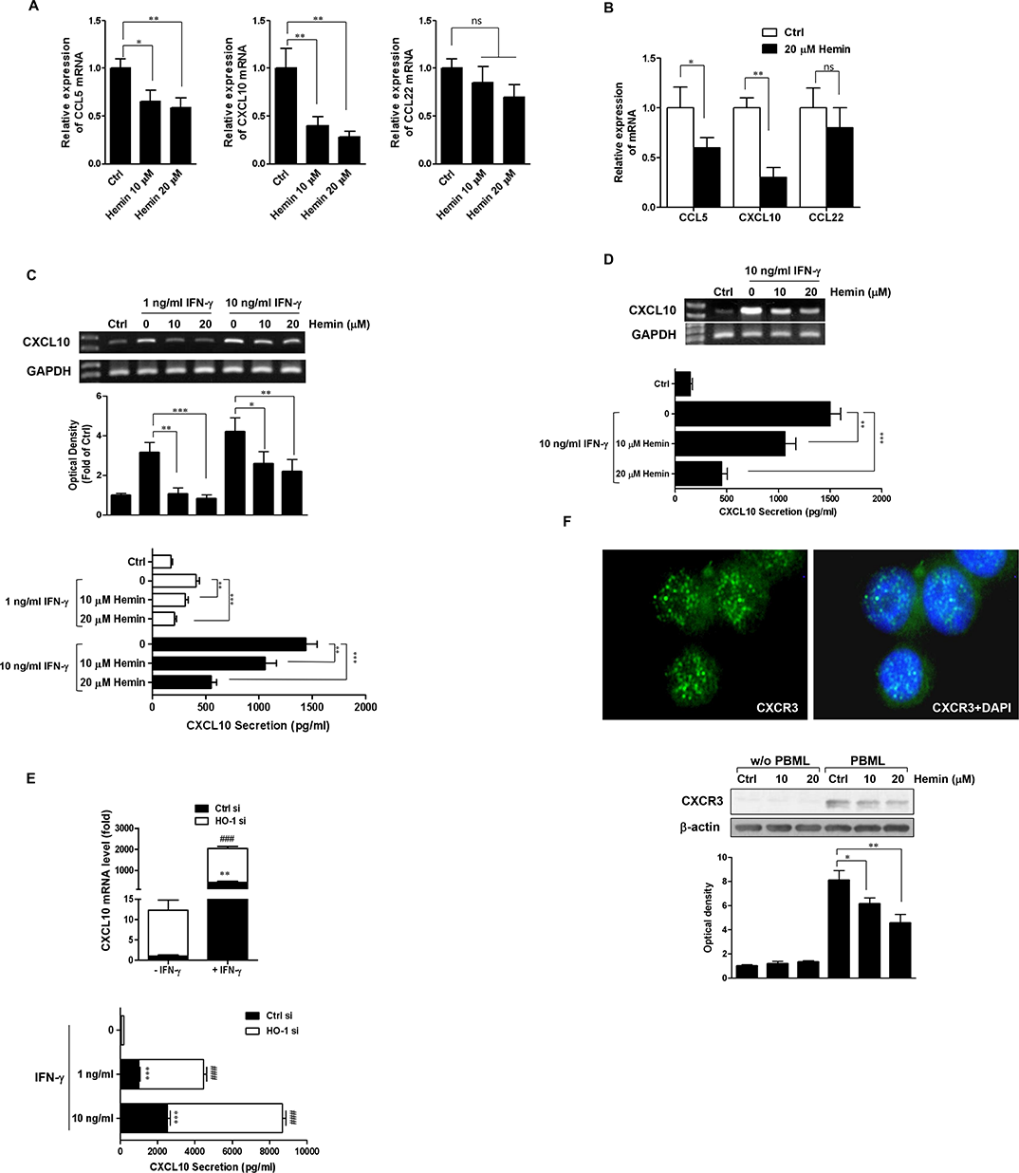
Effects of HO-1 on CXCL10 expression and secretion in CRC cells **A.** and **B.** The effect of HO-1 on CCL5, CXCL10, and CCL22 mRNA expression in HT-29 cells (A) and Caco-2 cells (B) Cells were treated with hemin for 16 h followed by CCL5, CXCL10, and CCL22 mRNA quantification by real time PCR. The values represent mRNA levels normalized to that of GAPDH mRNA. **C.** and **D.** The effect of HO-1 on CXCL10 mRNA expression and secretion in IFN-γ-stimulated HT-29 cells (C) and Caco-2 cells (D) Cells were pretreated with 10 and 20 μM hemin for 1 h and were stimulated with the indicated concentration of IFN-γ for an additional 16 h. CXCL10 mRNA was quantified by RT-PCR (each *upper panel*). CXCL10 secretion in cell culture supernatants was determined by ELISA (each *lower panel*). **E.** The effect of HO-1 on IFN-γ-induced CXCL10 expression in HT-29 cells. An HO-1 siRNA system was used to silence HO-1 mRNA in cells and to create a siRNA knockdown model in HT-29 cells. Cells were transfected with HO-1 siRNA for 24 h and were subsequently treated with IFN-γ for 16 h. CXCL10 mRNA was quantified by real time PCR (*upper panel*). CXCL10 secretion in the cell culture supernatants was determined by ELISA (*lower panel*). **F.** HO-1 induction of HT-29 cells decreased the adherence of CXCR3-positive PBMLs to HT-29 cells. The CXCR3 immunofluorescence assay was performed under same experimental condition as described in Figure 3. Following coculture, suspended PBMLs were removed and adhered PBMLs and HT-29 cells were fixed in paraformaldehyde. The *upper panel* is a representative image of CXCR3 expression in PBMLs added to the control (×400) and adhered PBMLs and HT-29 cells were harvested for the determination of CXCR3 protein expression by western blot analysis (*lower panel*). The results were repeated at least three independent experiments. The data are expressed as mean ± SD. ^*^P < 0.05, ^**^P < 0.01, and ^###^, ^***^P < 0.001.

The immune pattern of the tumor microenvironment for a large array of primary tumors is a major predictor of patient survival [19]. Because CRC cells can produce T_eff_- and T_reg_-recruiting chemokines, we used real-time PCR after hemin treatment of HT-29 cells to examine whether the expression of CCL5, CXCL10, and CCL22 is regulated by HO-1 in CRC cells. As shown in Figure 4A, the mRNA expression levels of the T_eff_-recruiting chemokines CCL5 and CXCL10 were significantly decreased by hemin in a dose-dependent manner. However, the mRNA expression of CCL22, a T_reg_-recruiting chemokine, was inhibited only slightly by hemin. Hemin treatment also caused similar effects on CCL5, CXCL10, and CCL22 mRNA expression in Caco-2 cells (Figure 4B).

Because IFN-γ affects the cancer immunoediting process, we examined whether the expression of CXCL10 is regulated by HO-1 in IFN-γ-stimulated CRC cells. Hemin dose-dependently reduced IFN-γ-induced CXCL10 mRNA expression and CXCL10 secretion in both HT-29 (Figure 4C) and Caco-2 cells (Figure 4D). To confirm the role of HO-1 in regulating the expression of CXCL10, HT-29 cells were transfected with siRNA against HO-1. To measure the CXCL10 transcript levels in the HO-1-silenced cells, we used real time PCR. As shown in Figure 4E (*upper panel*), even in the absence of IFN-γ stimulation, CXCL10 mRNA expression was upregulated in HT-29 cells transfected with HO-1-specific siRNA compared with that in cells transfected with control siRNA. Moreover, HO-1 siRNA transfection potentiated the IFN-γ-stimulated CXCL10 mRNA expression. The level of CXCL10 secretion paralleled that of its mRNA expression (Figure 4E, *lower panel*).

Our abovementioned data demonstrated that: (i) the increased HO-1 expression in CRC cells inhibited the adhesion of PBMLs to CRC cells (Figure 3) and (ii) the increased HO-1 expression in CRC cells reduced the expression and secretion of the T_eff_-recruiting chemokines CXCL10 (Figure 4A–4E). Therefore, we hypothesized that recruitment and subsequent adherence of PBMLs to CRC cells would increase the expression CXCR3, a receptor for CXCL10. Coculture of PBMLs and HT-29 cells produced a punctate (green fluorescence) expression of CXCR3 in PBMLs adhered to HT-29 cells (Figure 4F, *upper panel*). In the same experimental condition, immunoblotting showed an increase in the concentration of hemin concomitant with a decrease in CXCR3 expression in PBMLs adhered to HT-29 cells (Figure 4F, *lower panel*). These results indicated that the CXCL10-induced recruitment and adhesion of CXCR3-expressing PBMLs were decreased by the induction of HO-1 in CRC cells.

### HO-1 expression shows a trend toward a negative relationship with ICAM-1 and CXCL10 expression in xenograft tumors

**Figure 5:**
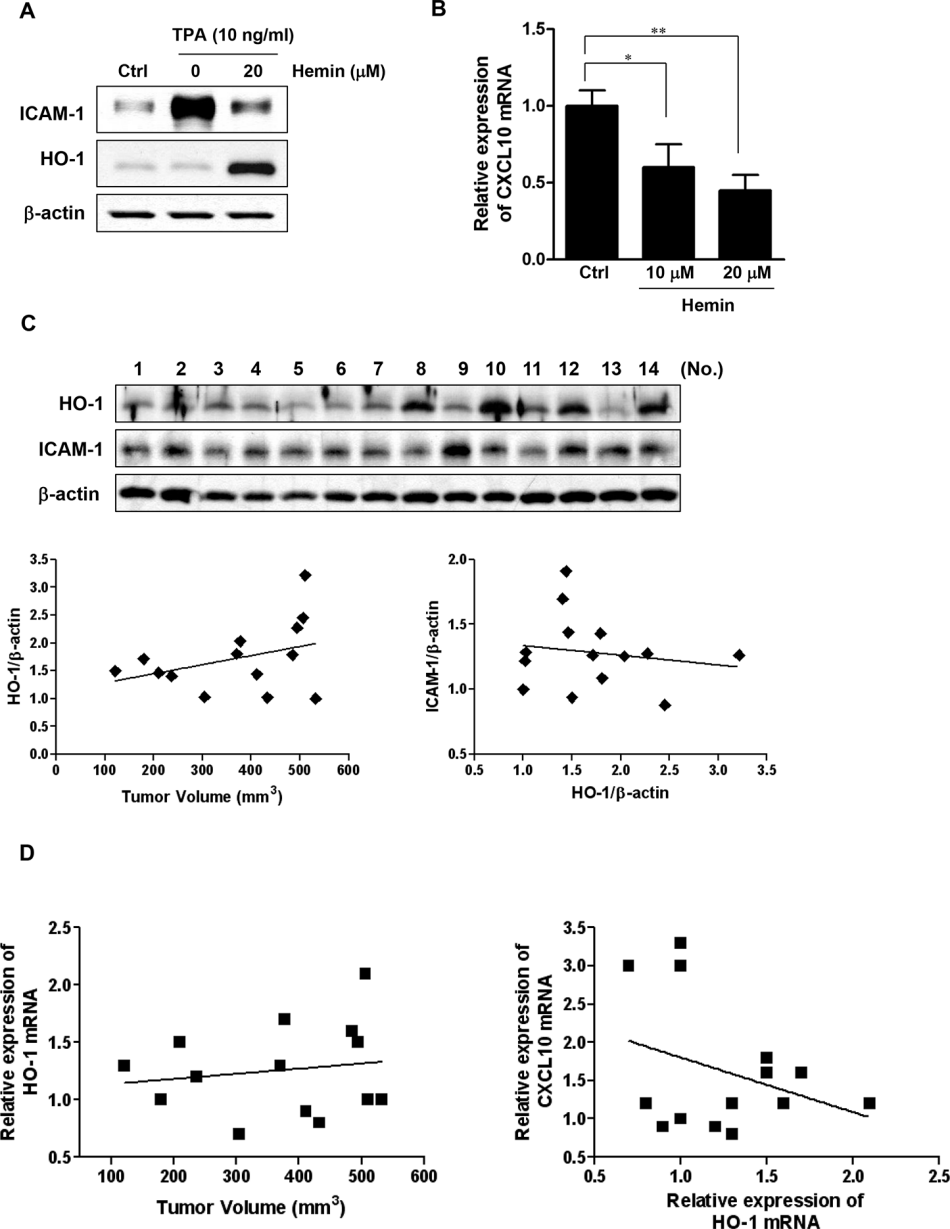
Expressions of HO-1, ICAM-1 and CXCL10 in xenograft tumors **A.** The effect of HO-1 on ICAM-1 protein expression in CT-26 cells. The cells were pretreated with 20 μM hemin for 1 h and were subsequently stimulated with 10 ng/ml TPA for an additional 24 h. The cells were harvested for ICAM-1 protein expression by western blot analysis. A representative immunoblot out of three independent experiments is shown. **B.** The effect of HO-1 on CXCL10 mRNA expression in CT-26 cells. Cells were treated with 10 and 20 μM hemin for 16 h followed by CXCL10 mRNA quantification by real time PCR. The values represent mRNA levels normalized to that of GAPDH mRNA. The results were repeated at least three independent experiments. The data are expressed as mean ± SD. ^*^P < 0.05 and ^**^P < 0.01. **C.** Western blot analysis of HO-1 and ICAM-1 expression in the enucleated tumors of the mice. β-Actin was used as a loading control (*upper panel*). Band densities were quantified and normalized to those of β-actin. Scatter plot trend lines were calculated using the standard linear regression analysis. The *lower panel* shows the relationship trend between tumor volume and HO-1 expression (*left*) and between HO-1 expression and ICAM-1 expression in the enucleated tumors (*right*). **D.** Each enucleated tumor was lysed and the RNA extracted for analysis of HO-1 and CXCL10 mRNA expression using real time PCR. These scatter plot trend lines show a relationship trend between tumor volume and HO-1 mRNA expression (*left*) and between HO-1 mRNA expression and CXCL10 mRNA expression (*right*).

The results described above indicated that HO-1 in CRC cells might affect immune surveillance, and we investigated further whether the tumor expression level of HO-1 could contribute to tumorigenicity. Because a syngeneic model can include an immune component, we induced tumor xenografts of mouse CT-26 colon cancer cells in mice. Similarly, hemin treatment of CT-26 cells suppressed TPA-induced ICAM-1 protein expression (Figure 5A) and significantly decreased CXCL10 mRNA expression in dose-dependent manner (Figure 5B). The mice were injected subcutaneously with CT-26 cells, and tumor growth was measured. The growth differed between the CT-26 xenograft tumors masses (384 ± 137 mm^3^ (mean ± SD) on day 28 after xenografting), despite injection of the same number of CT-26 cells (Supplementary Figure 1). Consistent with tumor volume, both HO-1 protein and ICAM-1 protein expression levels differed between enucleated tumors, as measured by western blot analysis (Figure 5C, *upper panel*). The relationships between tumor volume and protein expression were examined by scatter plot trend lines, which were calculated using standard linear regression analysis. The criterion for statistical significance was taken as *P* < 0.05. Even though no significant correlations were found, there were trends toward a positive relationship between tumor volume and HO-1 protein expression (Figure 5C, *lower left panel*), and toward a negative relationship between HO-1 protein and ICAM-1 protein expression (Figure 5C, *lower right panel*). There were also trends toward a positive relationship between tumor volume and HO-1 mRNA expression (Figure 5D, *left panel*), and toward a negative relationship between HO-1 mRNA expression and CXCL10 mRNA expression (Figure 5D, *right panel*). Although the correlations were not significant, these results suggest that HO-1 might be negatively related to ICAM-1 and CXCL10 expression in xenograft tumors.

## DISCUSSION

The expression of HO-1, which is often upregulated in CRC cells, is increased further in response to different types of treatment, including chemotherapy, irradiation, or photodynamic therapy [41–43]. Therefore, investigation into the role of HO-1 is vital for a better understanding of the regulation of tumor progression and for clinical practice. However, the results of previous studies on this topic remain inconsistent. Therefore, the objective of our study was to identify the role of HO-1 in the progression and metastatic potential of CRC cells. Most studies of the metastatic potential of HO-1 to date have focused on the antiapoptotic and/or proangiogenic properties of HO-1 even though there is no evidence that HO-1 directly regulates antitumor immunity in CRC cells.

A classical view of cancer progression is that genetic modifications may allow malignant cells to escape local and systemic immune control and, consequently, to invade and metastasize to distant organs. It has been reported that natural killer (NK) cells and T- and B-lymphocytes can recognize tumor cells as abnormal/foreign and then target them for destruction. An increased ICAM-1 cell surface expression may improve cancer cell recognition by the host effector cells [44, 45]. The incidence of liver metastasis in CRC is significantly lower in patients with ICAM-1-positive tumors than in those with ICAM-1-negative tumors [46].

In the present study, the HO-1 inducer hemin negatively regulated the expression of ICAM-1 in TPA-stimulated CRC cells. By contrast, ZnPP, an HO-1 inhibitor, increased ICAM-1 expression beyond that induced by TPA alone. These results suggest that HO-1 plays an important role in the regulation of ICAM-1 expression in TPA-induced CRC cell promotion. We also found that the adhesion of PBMLs to CRC cells and the subsequent PBML-mediated cytotoxicity against CRC cells were inhibited by HO-1 induction in CRC cells. Thus, the decreased adhesion of CRC cells to PBMLs might allow PBML less opportunity to lyse the target CRC cells. These results suggest that the increased expression of HO-1 in cancer cells might inhibit the immune defense function against host effector cells by modulating ICAM-1 expression.

We also found that HO-1 appeared to regulate ICAM-1 expression at the posttranscriptional level. HO-1 destabilized ICAM-1 mRNA in TPA-stimulated CRC cells. We also found evidence that the loss of stabilization of ICAM-1 mRNA by HO-1 was associated with an increase in the expression of TTP, an mRNA-binding protein. The HO-1 inducers hemin and CoPPIX significantly increased TTP mRNA and protein expression, whereas HO-1 inhibition did not induce TTP expression in CRC cells. This observation is consistent with the finding of Uddin *et al*., who reported that induction of HO-1 expression increased TTP level in macrophages [47]. However, in our present study, the HO-1-induced reduction in ICAM-1 level was similar in the TTP siRNA-transfected and control siRNA-transfected TPA-stimulated HT-29 cells (Supplementary Figure 2), which provides evidence that other components are involved in regulating ICAM-1 expression.

Many types of leukocytes, such as macrophages, dendritic cells, neutrophils, NK cells, and T- and B-lymphocytes, can infiltrate tumors [19]. Lymphocytes may play a dual role in tumor progression. Tumor-specific cytotoxic T lymphocytes can eliminate tumor cells by direct or antibody-dependent cytotoxicity. However, the recruitment of T_reg_ attenuates the immune response and may neutralize the cytotoxic effector cells. HO-1 expression in various immune cell populations plays an important role in modulating the immune reaction within the tumor [48, 49]. However, little is known about the effects of HO-1 expression in CRC cells on antitumor immunity in the tumor microenvironment. The present study demonstrated that CXCL10 expression and secretion were significantly decreased by increased HO-1 expression in CRC cells. Moreover, we found that the CXCL10-induced recruitment of CXCR3-expressing PBMLs was significantly decreased by the induction of HO-1 in CRC cells grown in a coculture with PBMLs. These results suggest that an increased expression of HO-1 in CRC cells might be regarded as an obstacle to an effective anticancer immune response.

In our *in vivo* study using a mouse xenograft model, there was a trend toward a positive relationship between tumor volume and HO-1 mRNA and protein expression in each enucleated tumor. By contrast, HO-1 protein and mRNA expression were negatively associated with ICAM-1 protein and CXCL10 mRNA expression, although the correlations were not significant. In reality, tumors are not simply masses of transformed epithelial cells, and tumor formation involves the coevolution of neoplastic cells together with the extracellular matrix and vascular endothelial, stromal, and immune cells. Although our *in vitro* work focused on epithelial CRC cells, the growth of xenograft tumors is not solely determined by transformed epithelial cells but also is affected by heterologous cells in the tumor microenvironment. Moreover, the expression of HO-1 in tumors was detected not only in transformed epithelial cells but also in macrophages and tumor-associated immunocytes. Even though the correlations were not significant, it is possible that HO-1, ICAM-1, and CXCL10 play roles in the tumor growth of CRC.

**Figure 6:**
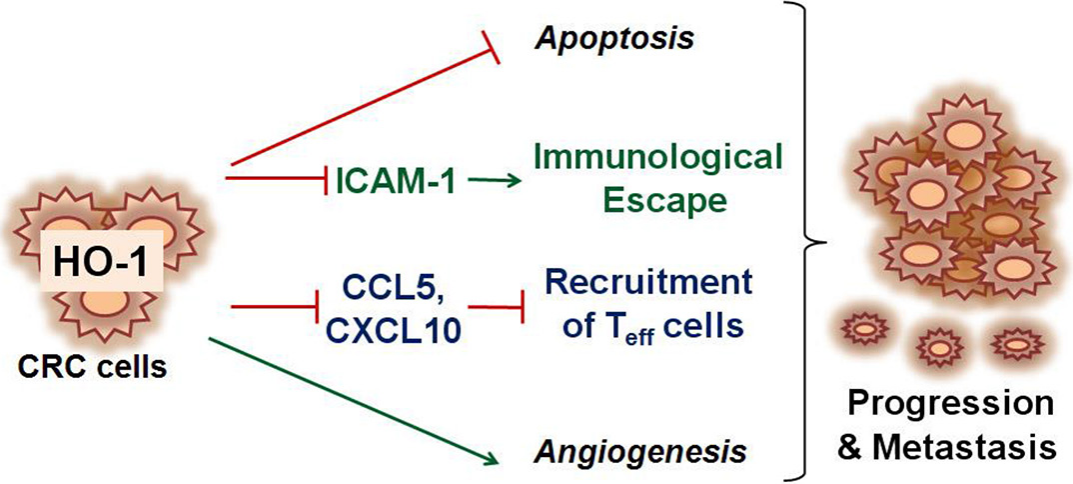
Schematic depiction of proposed mechanism associated with HO-1-mediated CRC progression and metastasis At present, most studies investigating the metastatic potential of HO-1 have focused on the antiapoptotic and/or proangiogenic properties of HO-1. The present results provide possible evidence that HO-1 directly regulates antitumor immunity in CRC cells. HO-1 reduces the expression of ICAM-1 and CXCL10, which in turn inhibit the adhesion and recruitment of T_eff_ cells to CRC cells and, consequently, inhibited T_eff_ cell-mediated cytotoxicity against CRC cells.

In summary, these results suggest that HO-1 in CRC cells is functionally linked to the regulation of tumor progression and metastasis in CRC. As shown in Figure 6, HO-1 reduces the expression of ICAM-1 and CXCL10, which in turn inhibits the adhesion and recruitment of CXCR3-expressing T_eff_ to CRC cells and consequently inhibits CXCR3-expressing T_eff_-mediated cytotoxicity against CRC cells. Development of successful immunotherapy for CRC will most likely require a multifaceted approach to boost tumor-specific effectors. The regulation of immunocytotoxicity by inhibiting HO-1 in CRC cells may provide a rationale for novel therapeutic approaches.

## MATERIALS AND METHODS

### Materials

Dulbecco's modified Eagle's medium (DMEM), fetal bovine serum (FBS), OPTI-modified Eagle's medium, 25% trypsin–ethylenediaminetetraacetic acid and penicillin–streptomycin solution were purchased from Gibco-BRL (Grand Island, NY, USA). TRIzol was purchased from Invitrogen (Carlsbad, CA, USA). RETROscript kit was obtained from Ambion (Austin, TX, USA). SYBR Green PCR Core Reagents were purchased from TAKARA (Warrington, Japan). Dithiothreitol (DTT), 3-(4, 5-dimethylthiazol-2-yl)-2, 5-diphenyltetrazolium bromide (MTT), the PKH67 green fluorescent cell linker kit, HISTOPAQUE^®^-1077, 0.4% trypan blue solution, 12-*O*-tetradecanoylphorbol-13-acetate (TPA), hemin, cobalt protoporphyrin IX (CoPPIX), zinc protoporphyrin (ZnPP), actinomycin D (Act D), Fluoroshield^™^ with DAPI, a primary antibody against β-actin and fluorescein isothiocyanate (FITC)-conjugated anti-rabbit immunoglobulin were purchased from Sigma Chemical Co. (St Louis, MO, USA). The NF-κBp65 transcription factor enzyme-linked immunosorbent assay (ELISA) kit was purchased from Cayman Chemical Company (Ann Arbor, MI, USA). Primary antibodies against ICAM-1, p65, HO-1 and tristetraprolin (TTP) were purchased from Santa Cruz Biotechnology (Santa Cruz, CA, USA). Antibodies against TATA-binding protein (TBP, a nuclear protein loading control) and CXCR3 were from Abcam (Cambridge, UK). Horseradish peroxidase (HRP)-conjugated secondary antibodies were obtained from Santa Cruz Biotechnology (Santa Cruz, CA, USA). An neutralizing antibody against the human ICAM-1 antibody, recombinant IFN-γ, and the human CXCL10/IP-10 Quantikine ELISA Kit were purchased from R&D Systems (Minneapolis, MN, USA). The human-specific HO-1 small interfering RNA (siRNA) and non-targeting siRNA were purchased from Santa Cruz Biotechnology (Santa Cruz, CA, USA). The transfection reagent, Dharmacon's Dharmafect, was obtained from Dharmacon RNA Technologies (Lafayette, CO, USA).

### Cell culture

The human CRC cell lines HT-29 and Caco-2 were purchased from the American Type Culture Collection (Manassas, VA, USA) and were cultured in DMEM supplemented with 10% FBS, 100 U/ml penicillin and 100 μg/ml streptomycin. Cells were synchronized in DMEM containing 0.5% serum before treatments.

### Reverse transcription PCR (RT-PCR) and quantitative real-time PCR

For the measurement of mRNA levels of the genes of interest, total RNAs were isolated using TRIzol. RNA samples were reverse transcribed using the RETROscript kit with random decamers as primers. RT-PCR was performed in the presence of 1.5 mM MgCl_2_ at the following temperatures and times: 94°C for 1 min, 60°C for 1 min and 72°C for 1 min. The following primers were used: ICAM-1, 5′- CCGGAAGGTGTATGAACTG-3′ (forward) and 5′- TCCATGGTGATCTCTCCTC-3′ (reverse); CXCL10, 5′- CTGAAAGCAGTTAGCAAGGAAAG-3′ (forward) and 5′-GATGGGAAAGGTGAGGGAAATA-3′ (reverse); glyceraldehyde 3-phosphate dehydrogenase (GAPDH), 5′-GATTCCACCCATGGCAAATTC-3′ (forward) and 5′- CATGAGTCCTTCCACGATAC-3′ (reverse).

Real-time PCR amplification was performed using SYBR Green PCR Core Reagents. The amount of target mRNA was determined using the comparative threshold (Ct) method by normalizing the target mRNA Ct values to those of GAPDH (ΔCt). Statistical analysis of real-time PCR data was performed using ΔCt values. The following primer sequences were used in the *in vitro* study: ICAM-1, 5′-ATAACCGCCAGCGGAAGATCAAGA-3′ (forward) and 5′-CGTGGCTTGTGTGTTCGGTTTCAT-3′ (reverse); TTP, 5′-CCAAGTGTGCAAGCTCAGTAT-3′ (forward) and 5′- GGGTTCATTGCCTCCCTTAAA-3′ (reverse); CCL5, 5′-TGCCCACATCAAGGAGTATTT-3′ (forward) and 5′-GATGTACTCCCGAACCCATTT-3′ (reverse); CCL22, 5′-GCCAGCCTGGAAACTTAC T-3′ (forward) and 5′-CCTCCCAAAGTCCTGAGATTAC-3′ (reverse); CXCL10, 5′-GTAATAACTCTACCCTGGCACT ATAA-3′ (forward) and 5′-GATGGGAAAGGTGAGGGA AATA-3′ (reverse); HO-1, 5′-TCAGGCAGAGGG TGATAGAA-3′ (forward) and 5′-GCTCCTGCAA CTCCTCAAA-3′ (reverse); GAPDH, 5′-GGAGAAA CCTGCCAAGTAT GA-3′ (forward) and 5′-TCCT CAGTGTAGCCCA AGA-3′ (reverse). The primer sequences used in the mouse xenograft study were as follows: mHO-1, 5′-CTCCCTGTGTTTCCTTTCTCTC-3′ (forward) and 5′- GCTGCTGGTTTCAAAGTTCAG-3′ (reverse); mCXCL10, 5′- AGTAACTGCCGAAGCAAGA A-3′ (forward) and 5′-GCACCTCCACATA GCTTAC A-3′ (reverse); mGAPDH, 5′- GGAGA AACCTGCCAAGT ATGA-3′ (forward) and 5′- TCCTCAGTGTAGCCCAA GA-3′ (reverse).

### Preparation of cytosolic and nuclear extracts

Nuclear extracts were prepared as previously described [50]. Briefly, cells were scraped, washed with phosphate-buffered saline (PBS, pH 7.4), resuspended in hypotonic buffer containing 10 mM HEPES (pH 7.9), 10 mM KCl, 0.5 mM DTT, 0.2 mM PMSF, 1.5 mM MgCl_2_, and 1.2% Nonidet P-40 (Sigma; St Louis, MO, USA), and allowed to swell on ice for 10 min. Lysates were separated by centrifugation at 3300 × *g* for 5 min at 4°C. The supernatant represented the cytosolic extract. The nuclear pellets were extracted in nuclear extraction buffer containing 20 mM HEPES (pH 7.9), 0.4 M NaCl, 1.5 mM MgCl_2_, 0.2 mM EDTA, 25% glycerol, 0.5 mM PMSF, and 0.5 mM DTT for 30 min on ice, and centrifuged at 12, 000 × *g* for 30 min. The supernatant was used as the nuclear extract and stored at −70°C until use. The protein concentration of the nuclear extracts was determined using the Bio-Rad protein assay dye (Bradford) Reagent (Bio-Rad Laboratories, Hercules, CA, USA).

### NFκBp65 DNA binding assay

The activation of NF-κBp65 was assessed by an enzymatic immunoassay. The nuclear extract samples were used for the analysis of NF-κB DNA binding using the NF-κBp65 transcription factor ELISA kit according to the manufacturer's instructions. Briefly, nuclear extracts were incubated in the oligonucleotide-coated wells in which the oligonucleotide sequence contained the NF-κB response element consensus-binding site. After washing, samples were incubated with a specific primary antibody directed against NF-κBp65. A secondary HRP-conjugated antibody was added to provide a sensitive colorimetric readout at 450 nm.

### Heme oxygenase activity assay

HO activity was measured by bilirubin generation as described previously (Ref). Briefly, microsomes from harvested cells were added to a reaction mixture containing NADPH, glucose-6-phosphate dehydrogenase, rat liver cytosol as a source of biliverdin reductase, and the substrate hemin. The reaction mixture was incubated in the dark 37°C for 1 h and terminated by the addition of chloroform. After being vigorously vortexed and centrifuged, the amount of extracted bilirubin in the chloroform layer was determined by measuring the difference in absorbance between 464 and 530 nm, with a molar extinction coefficient of 40 mM − 1 cm − 1. HO activity was expressed as picomoles of bilirubin formed per hour per milligram protein.

### Western blot analysis

Protein lysates were separated by 10% sodium dodecyl sulfate polyacrylamide gel electrophoresis (SDS-PAGE) and subsequently electrotransferred onto nitrocellulose membranes (Amersham Pharmacia Biotech Inc., Piscataway, NJ, USA). Membranes were blocked with 5% non-fat milk for 1 h at room temperature. The blocked membranes were subsequently incubated with primary antibodies. Immunoreactive bands were detected by incubating the membranes with HRP-conjugated secondary antibodies and chemiluminescence reagents to quantify the relative expression levels.

### CRC cells adhesion assay to FBS-coated plastic wells

Prior to the adhesion assay, HT-29 cells were fluorescence-labeled using the PKH67 green fluorescent cell linker kit according to the manufacturer's instructions. Briefly, the whole procedure was performed at 20–25°C. Cells (1 × 10^7^) were washed with serum-free DMEM. The cell suspension was centrifuged at 400 × *g* for 5 min to produce a cell pellet and the supernatant was removed. A 1 ml portion of Diluent C was added to resuspend the cells. The dye was diluted with Diluent C immediately before staining. The cells in Diluent C were added rapidly to 1 ml of this dye solution. The cells and dye were mixed by gentle pipetting. The mixture was incubated at 20–25°C for 5 min with periodic mixing. The staining process was stopped by adding an equal volume of FBS and incubating for 1 min. The stained cells were diluted with an equal volume of complete culture medium, centrifuged at 400 × *g* for 10 min and washed at least three times. Then, the cells were resuspended in fresh complete medium. Fluorescent-labeled HT-29 were pre-incubated with hemin for 30 min and then plated on FBS-coated 24-well plates for 4 h in the presence of TPA at 37°C under 5% CO_2_. After incubation, non-adherent HT-29 cells were removed and adherent cells were counted using a fluorescence image analyzer (JuLI™ Fluorescent analyzer, Bull-dog Bio, Inc., Rochester, NY, USA). The adherent cells of each well were quantified as the mean number of cells in five high-power fields.

### PBML adhesion assay to CRC cells

Human peripheral blood mononuclear lymphocytes (PBMLs) were separated from peripheral blood of healthy donors using Ficoll HISTOPAQUE^®^-1077 gradient centrifugation at room temperature. Cells were collected, washed and resuspended in DMEM with 10% FBS. The cell count and viability was determined using Trypan blue solution. Cell preparations of more than 95% viability were used for the experiments. To evaluate PBML adhesion to cancer cells, CRC cells (1 × 10^5^/well) were incubated with TPA alone, TPA and hemin, or TPA and anti-ICAM-1 neutralizing antibody for 24 h in 96-well microplates. After washing of the plates with PBS, PBMLs (1 × 10^6^/well) were added to allow attachment to CRC cells during 1 h at 37°C. The binding of PBMLs was quantified using a colorimetric assay with MTT [44, 45]. The reduction of MTT to formazan was assessed in an ELISA plate reader. The ratio of the total PBMLs that adhered to CRC cells was calculated as follows: adhesion ratio = (OD of experimental wells – OD of CRC cell wells)/OD of total PBML wells. Data expressed as % adhesion compared to TPA only-treated group which was set as 100%.

### PBML-mediated cytotoxicity assay of CRC cells

The lactate dehydrogenase (LDH) assay was used to assess cell cytotoxicity [44, 45]. After incubation of CRC cells with TPA alone, TPA and hemin, or TPA and anti-ICAM-1 neutralizing antibody for 24 h, PBMLs were added and incubated at 37°C for 16 h. LDH leakage into the medium was quantified by an Autodry Chemistry Analyzer (SPOTCHEM SP4410, Arkray, Japan). Three replicate wells for spontaneous (CRC cell monoculture in DMEM with 10% FBS) and maximum release (CRC cell monoculture in DMEM with 1% Triton X-100) were measured in parallel. The ratio of total lysed cancer cells was calculated as follows: cytotoxicity ratio = (experimental LDH − PBMLs spontaneous LDH − CRC cells spontaneous LDH)/(CRC cells maximum LDH − CRC cells spontaneous LDH). Data expressed as % cytotoxicity compared to TPA only-treated group which was set as 100%.

### siRNA transfection

Approximately 1 × 10^6^ cells were transfected with HO-1 siRNA or non-targeting siRNA (used as a control) using Dharmacon's Dharmafect transfection reagent, according to the manufacturer's instructions. Cells were collected 48 h after transfection and used for subsequent experiments.

### CXCL10 ELISA

CXCL10 protein secretion was measured by ELISA according to the manufacturer's protocol. A microplate reader set at 450 nm was used to measure the concentration of CXCL10 from the supernatant with reference to the CXCL10 standard curve.

### CXCR3 immunocytochemistry

Cells were fixed in 4% formaldehyde for 10 min and incubated in 1% bovine serum albumin (BSA)/0.1% Tween/PBS for 1 h to permeabilize the cells and block non-specific protein-protein interactions. The cells were subsequently incubated with the anti-CXCR3 antibody (diluted 1/1000) overnight at 4°C. To visualize the labeled sites, the slides were washed and then covered with FITC-conjugated anti-rabbit immunoglobulin (diluted 1/200) for 2 h at room temperature. DAPI was used to stain the cell nuclei. The fluorescence was visualized using a Nikon microscope (Model UFX-IIA; Nikon, Melville, NY, USA).

### Mouse tumor xenograft model

All experimental procedures involving animals were carried out in strict accordance with the Guide for the Care and Use of Laboratory Animals (National Institutes of Health, USA); all experiments were approved by the Animal Studies Ethics Committee of Wonkwang University. Male BALB/c mice (seven to eight weeks old) were purchased from Orient Animal Breeding Center (Sungnam, Korea). All animals were housed in wire cages at 20 to 22 °C and 50 ± 10% humidity, fed standard laboratory chow (Orient Animal Breeding Center, Sungnam, Korea) and allowed water *ad libitum*. BALB/c mouse strain CT-26 mouse colon cancer cells (5 × 10^6^ cells; American Type Culture Collection, Manassas, VA, USA) suspended in 0.1 ml PBS were subcutaneously injected into the right flanks of the mice. Tumor size was measured weekly using calipers, and tumor volumes were calculated according to the following formula: (length × width^2^)/2. Mice were sacrificed four weeks after tumor cell inoculation and each tumor was removed and subsequently prepared for real-time PCR and western blot analysis.

### Statistical analysis

Data were analyzed using Student's *t*-test when appropriate. A one-way analysis of variance (ANOVA) and Tukey's multiple comparison tests were applied when comparing more than three means. In xenograft study, standard linear regression analysis based on minimizing the sum of squares of the vertical distances of the points from the line. Statistical analysis was performed using the GraphPad Prism 5 software (GraphPad Software, San Diego, CA, USA).

## SUPPLEMENTARY FIGURES


