# Supplementary material for: Heme oxygenase-1 promotes tumor progression and metastasis of colorectal carcinoma cells by inhibiting antitumor immunity
Source: Oncotarget. 2015 May 28;6(23):19792–806. doi: 10.18632/oncotarget.4075 (PMC4637321; doi:10.18632/oncotarget.4075)
Supplement: Supplementary file 1 [file oncotarget-06-19792-s001.pdf]

SUPPLEMENTARY FIGURES

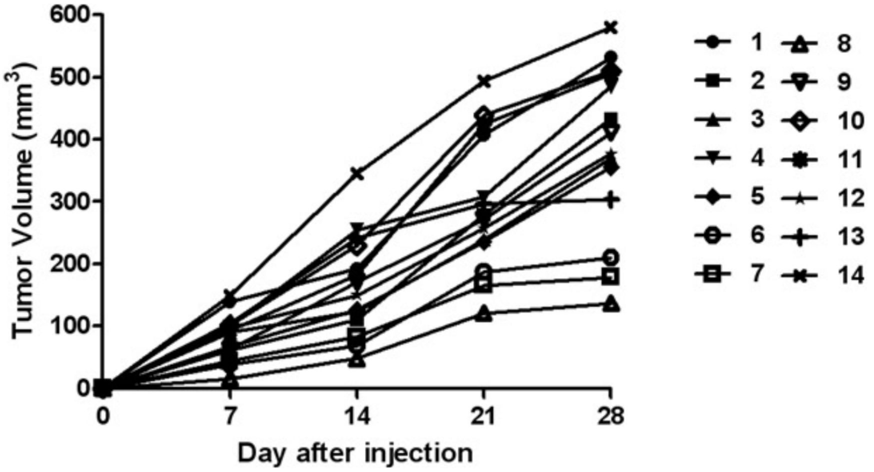

Supplementary Figure 1: Tumor growth of CT-26 mouse colon carcinoma cells inoculated into BALB/c mice. CT-26 tumors were established in BALB/c male mice (*n* = 14) by a subcutaneously injection of CT-26 cells into the right flank. Tumor volumes were monitored weekly.

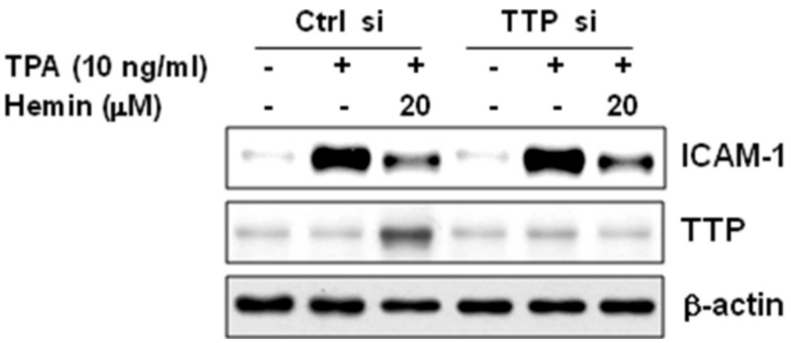

Supplementary Figure 2: The effect of TTP silencing on the hemin-mediated reduction of ICAM-1 in TPA-induced HT-29 cells. HT-29 cells were transfected with siRNA against TTP (TTP si) or control siRNA (Ctrl si). After pretreatment with 20 µM hemin, and then stimulated with 10 ng/ml TPA for an additional 24 h. The expression levels of ICAM-1 and TTP were analyzed by western blot analysis. A representative immunoblot out of three independent experiments is shown.
